# Functional trait dataset of benthic macroinvertebrates in South Korean streams

**DOI:** 10.1038/s41597-023-02678-y

**Published:** 2023-11-28

**Authors:** Sagar Adhurya, Da-Yeong Lee, Dae-Seong Lee, Young-Seuk Park

**Affiliations:** Ecology and Ecological Informatics Laboratory, Department of Biology, College of Science, Kyung Hee University, Seoul, 02447 Republic of Korea

**Keywords:** Biodiversity, Community ecology

## Abstract

Functional traits are the result of evolution and adaptation, providing important ecological insights into how organisms interact with their environment. Benthic macroinvertebrates, in particular, have garnered attention as biomonitoring indicators for freshwater ecosystems. This study presents a functional trait dataset for benthic macroinvertebrates, comprising 447 taxa (393 at genus level, 53 at family level and one at class level) from five phyla (Annelida, Arthropoda, Mollusca, Nematomorpha, and Platyhelmenthes), categorized into nine traits related to life history, morphology, and habit. To account for variation in available trait information, we assigned confidence levels to each taxon and functional trait based on the level of evidence using fuzzy coding. Our dataset provides an important resource for understanding the ecology of benthic macroinvertebrates in South Korea, serving as a valuable baseline dataset for studying their biodiversity, conservation, and biomonitoring in freshwater ecosystems.

## Background & Summary

‘Functional trait’ are any characteristics of an organism, such as morphological, physiological, biochemical, behavioural, and phenological traits, that influence its fitness or survival^1^. It aids in understanding a species’ ecological adaptation to its environment and the community’s response to eco-environmental change^2,3^. It is considered a currency of functional ecology to assess the functional properties of ecological communities^4,5^. It is used to measure functional diversity, which helps to understand how an ecosystem functions^6^.

Functional traits bridge the gap between ecology and evolution, providing insight into various scientific questions related to biogeography, ecosystem health, and conservation^7–10^. Furthermore, the functional trait-based approach to understand ecology enables global comparisons of ecological responses, despite taxonomic differences in species assemblages^8,11^. Given immense importance of the functional traits, there is a growing demand for trait datasets to progress the field of functional ecology. However, collecting trait data requires significant cost and time investment, resulting in a limited number of trait datasets covering only a few taxa and biogeographic regions.

The diversity of benthic macroinvertebrates and their functional traits make them an ideal model group for biomonitoring freshwater ecosystems^12^, as they have an intermediate lifespan and a diverse array of functional traits that help measure changes in ecosystems^13,14^. Despite the immense importance of trait data for freshwater benthic macroinvertebrates, only a few datasets covering a small biogeographic portion of the globe exist, such as CESTES (Mediterranean rivers, Catalonia, Spain; Segura River basin, Spain; Ebro river, Mediterranee, Spain; Ponds, agricultural areas, Brie, Seine-et-Marne, France; Wu Stream, central Taiwan; and Ponds, 200-ha section of the Yale-Myers Research Station in Union, Connecticut, USA)^15^; European aquatic macroinvertebrates dispersal related trait dataset^16^, European freshwater organisms trait dataset^17^; stream macroinvertebrates of Han river basin, China^18^, lotic insects of North America^19,20^ and freshwater macroinvertebrates of New Zealand^21^. This limited number of datasets for a small part of the world underscores the need for a worldwide aquatic macroinvertebrate data collection program to develop a global dataset. Such a dataset would help fill a significant gap in functional ecology and enable a better understanding of the consequences of environmental change due to different drivers, such as climate change and anthropogenic activities, on benthic macroinvertebrates worldwide.

In this study, we developed a functional trait dataset for benthic macroinvertebrates in South Korean streams. The dataset consists of functional traits of 447 taxa. The dataset was constructed using occurrence data of macroinvertebrates collected from 3032 locations throughout South Korea as part of the National Aquatic Ecological Monitoring Program (NAEMP) from 2008 to 2021. We considered nine traits across three categories, namely life history, morphology, and habit, and obtained trait data from various literature sources. Besides fulfilling the gap in macroinvertebrate trait data, the dataset can be utilized for various scientific studies to understand the autecology of benthic macroinvertebrates in Asian streams, including Korea, along with its further comparison to global counterparts, biomonitoring and conservation planning.

## Methods

### Taxonomic and geographical coverage

**Fig. 1:**
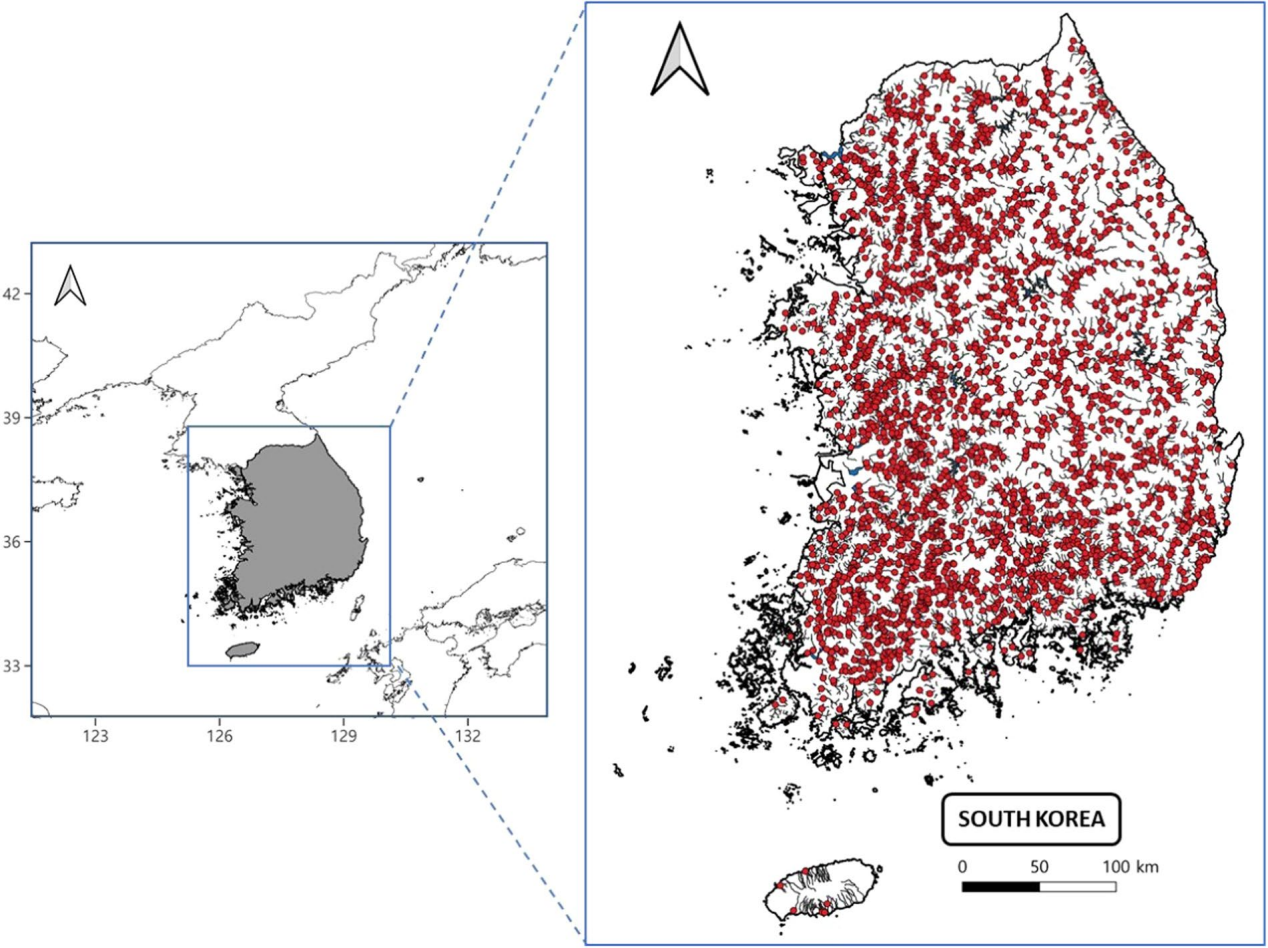
Location of the survey sites across South Korea for the biomonitoring in NAEMP (on the right). On the left, the relative position of South Korea on the map of northeast Asia showing the Korean peninsula is indicated.

The dataset covered almost all streams of South Korea (Fig. 1) and was compiled from biomonitoring data available on the National Institute of Environmental Research (NIER) website (https://water.nier.go.kr/web/bioMeasure?pMENU_NO=586). This data was collected collaboratively according to NIER guidelines under the NAEMP from 2008 to 2021, covering 3032 sampling locations^22^. Additionally, eight additional genera were included from another published article^23^.

### Taxonomy and systematics

The compiled data includes 908 macroinvertebrate taxa. However, due to the unavailability of species-level trait data for many species, we established the taxonomic resolution of our dataset at the genus level, resulting in 455 genera. In some instances, the specimens were identified only up to the subfamily (e.g., Acentropinae), family (e.g., Saldidae), or class level (e.g. Collembola) in the original dataset. We used “genera” to refer to the lowest identifiable level in our dataset. These genera were classified according to the GBIF backbone taxonomy into four taxonomic hierarchies: Family, Order, Class, and Phylum. We updated some genus names to match those used in GBIF and corrected seven inconsistent genera, resulting in a final dataset with the data for 393 taxa at genus level, 53 taxa at family level and one taxon at class level. We removed two genera due to their synonymy with existing genera, four genera for spelling errors, and one genus that was not a macroinvertebrate.

### Functional traits

Based on available data, we selected nine functional traits and sorted them into three categories: *life history*, *morphology* and *habit* (Table 1). These traits were selected based on existing literature and data availability. While some traits such as fecundity, environmental tolerance, synchronization of emergence, resistance form, and the propensity of drift have been excluded due to data scarcity, we intend to expand our dataset in the future as more data becomes available.

**Table 1:** Summary of the traits used in this dataset.

Category	Trait name	Modality	Abbreviations
Life History	Voltinism	Semivoltine (<1 generation/year)	V1
		Univoltine (1 genereation/year)	V2
		Multivoltine (>1generation/year)	V3
	Life span	≤1 year	L1
		>1 year	L2
	Aquatic stages	Egg & Larva	AS1
		Fully aquatic	AS2
Morphology	Maximum size	Small (<9 mm)	MS1
		Medium (9 to 16 mm)	MS2
		Large (>16 mm)	MS3
	Respiratory organ	Tegument	R1
		Gill	R2
		Aerial (also including plastron)	R3
	Shape	Streamlined (includes flat & fusiform)	S1
		Non-streamlined (includes cylindrical, round and bluff)	S2
	Armouring	None	AR1
		Weak	AR2
		Strong	AR3
Habit	Locomotion	Swimmer/Planktonic/Skater/Flier	LO1
		Crawler/Climber/Sprawler	LO2
		Burrower/Interstitial	LO3
		Clinger	LO4
	Functional feeding group	Collectors-gatherer	F1
		Collector-filterer	F2
		Scraper/Grazer (herbivore)	F3
		Shredder	F4
		Predator (including piercer)	F5
		Parasites	F6

*Life history* contains three traits, i.e., *voltinism*, *life span* and *aquatic stages*. *Voltinism* indicates the number of generations per year^24^, which positively impacts intraspecific size structure variation and negatively affects intraspecific competition & carnivory^25,26^. *Life span* is the average life cycle duration linked to a species’ reproductive potential^27^. Generally, species with shorter life spans are more tolerant to disturbance^28^. *Aquatic stages* indicate dispersal capability, and non-aquatic adults with flying ability typically have higher dispersal capability^29,30^.

*Morphology* encompasses four traits: *maximum size*, *respiratory organ*, *shape* and *armouring*. *Maximum size* is positively related to fecundity^31^, trophic level^32,33^, and mobility^34^ in aquatic macroinvertebrates. The *respiratory organ* denotes how an organism adapts to various environmental conditions and its oxygen tolerance^35^. *Shape* constrains mobility and reflects an organism’s adaptation to differing water flow levels^36,37^, while *armouring* conveys its capacity to withstand mechanical and environmental stresses^38,39^.

*Habit* contains two traits: *locomotion* and *functional feeding habit*. *Locomotion* mode and substrate relation affect microhabitat selection^40^ and ecosystem resilience by connecting habitats^41^. In contrast, *functional feeding groups* provide insights into trophic dynamics^42^ and response to perturbations^43^.

### Trait information collection

Initially, we searched macroinvertebrate datasets^15–21^ to gather trait information for various genera. Despite our efforts, trait information for numerous novel genera remained incomplete. We turned to Korean books^44,45^ and web resources^46,47^ to fill these gaps, and then we scoured journal articles and books. Since Korea, Japan, and China share similar species composition, we preferred trait information sourced from species in these regions. Additionally, we consulted numerous websites, as listed in the attached dataset’s reference sheet. Unfortunately, for many genera, we were unable to locate trait information. In such cases, we used trait information for higher taxonomic categories marked with a fuzzy code, with some exceptions outlined in the next section.

### Fuzzy coding of the modalities

We utilized a fuzzy coding framework to express the confidence level in trait modalities within our dataset, a method commonly employed in similar datasets^15,16,21^. We used three levels of fuzzy coding in this dataset where 0, 1, 2 and 3 indicate absence, low level, moderate level and high level of confidence, respectively. We established rules for the fuzzy coding process as follows:

1. If no reference supports the presence of a particular trait for a genus, it is denoted with 0.
2. If only one reference indicates a particular trait modality and there is no evidence about other trait modalities of a trait, then it is denoted as 2.
3. If multiple references indicate a particular modality without evidence for other modalities, it is coded as 3.
4. If the majority of evidence supports one modality while a single reference indicates the presence of another, the former is coded as 3, and the latter is coded as 1.
5. If the evidence for two different modalities is equal, both modalities are coded as 2, unless all references indicate the presence of both modalities, in which case they are coded as 3.
6. If one modality has the most evidence, while another has less, and a third has the least, they are coded as 3, 2, and 1, respectively. There can be a case where there is no evidence for the third. It can be coded as 3,2 and 0 respectively.
7. If a modality is inferred from a higher taxonomic level, such as a family, order, class, or phylum, it is coded with less confidence, unless it applies to all members of that group, in which case it is coded as 3 (e.g., hair in mammals).
8. In some cases, trait modalities were inferred from other databases, some of which used fuzzy coding. In this case, fuzzy codes across all modalities are summed up and then individual references are added as a single score against each modality. Then the fuzzy codes are inferred as per the above rules (Table 2).

**Table 2:** Table describing rule 8 of fuzzy coding considering databases with fuzzy code and references without fuzzy code.

Reference	Modality 1	Modality 2	Modality 3
1 (fuzzy code)	3	2	0
2 (fuzzy code)	2	0	0
3 (single reference)	-	1	-
4 (single reference)	1	-	-
Sum of score	6	3	0
Resulting fuzzy code	3	2	0

By applying these rules, our fuzzy coding framework provides a flexible and consistent approach to representing the confidence in trait modalities within our dataset.

## Data Records

### Dataset

The dataset^48^ is available in the latest Excel Workbook (*.xlsx) format and includes five sheets: Trait dataset, Datakey, Reference, Source reference and Korean endemics. The first sheet contains taxon names, lowest taxonomic ranks, and classifications in the first eight columns, while the remaining columns have trait modalities and references supporting the fuzzy coding of each modality (Table 3). Trait modalities are represented by abbreviations, with explanations available in the second sheet (Datakey). References in the Trait Dataset are identified by reference numbers, with corresponding details available in the third sheet (Reference). The fourth sheet contains source references in the large databases cited in the ‘Reference’ sheet. It has four columns. The first column indicates taxon name, second column indicates trait name, third column indicates the references to the database cited in ‘Reference’ sheet and the last column indicates the actual source reference. The last sheet represents a list of Korean endemic species those are included in this work.

**Table 3:** Structure of the Trait Dataset sheet. Here M1-M3 indicate three modalities of an arbitrary Trait A.

Sl. No.	Taxon name	Lowest taxonomic rank	Classification					Trait A			
			Phylum	Class	Order	Family	Genus	M1	M2	M3	Ref. No.
1	Erpobdella	Genus	Annelida	Clitellata	Arhynchobdellida	Erpobdellidae	Erpobdella	2	3	1	1,2,4
2	Hirudinidae	Family	Annelida	Clitellata	Arhynchobdellida	Hirudinidae	-	2	0	0	5
3	Hirudo	Genus	Annelida	Clitellata	Arhynchobdellida	Hirudinidae	Hirudo	3	0	0	2,5,6
…	…	…	…	…	…	…	…	…	…	…	…
29	Collembola	Class	Arthropoda	Collembola				0	0	2	8
30	Chrysomelidae	Family	Arthropoda	Insecta	Coleoptera	Chrysomelidae	-	0	0	1	29
…	…	…	…	…	…	…	…	…	…	…	…
438	Dugesia	Genus	Platyhelminthes	-	Tricladida	Dugesiidae	Dugesia	0	3	0	438
439	Sphalloplana	Genus	Platyhelminthes	-	Tricladida	Kenkiidae	Sphalloplana	0	1	0	439
440	Phagocata	Genus	Platyhelminthes	-	Tricladida	Planariidae	Phagocata	0	2	0	2

### Data summary

**Fig. 2:**
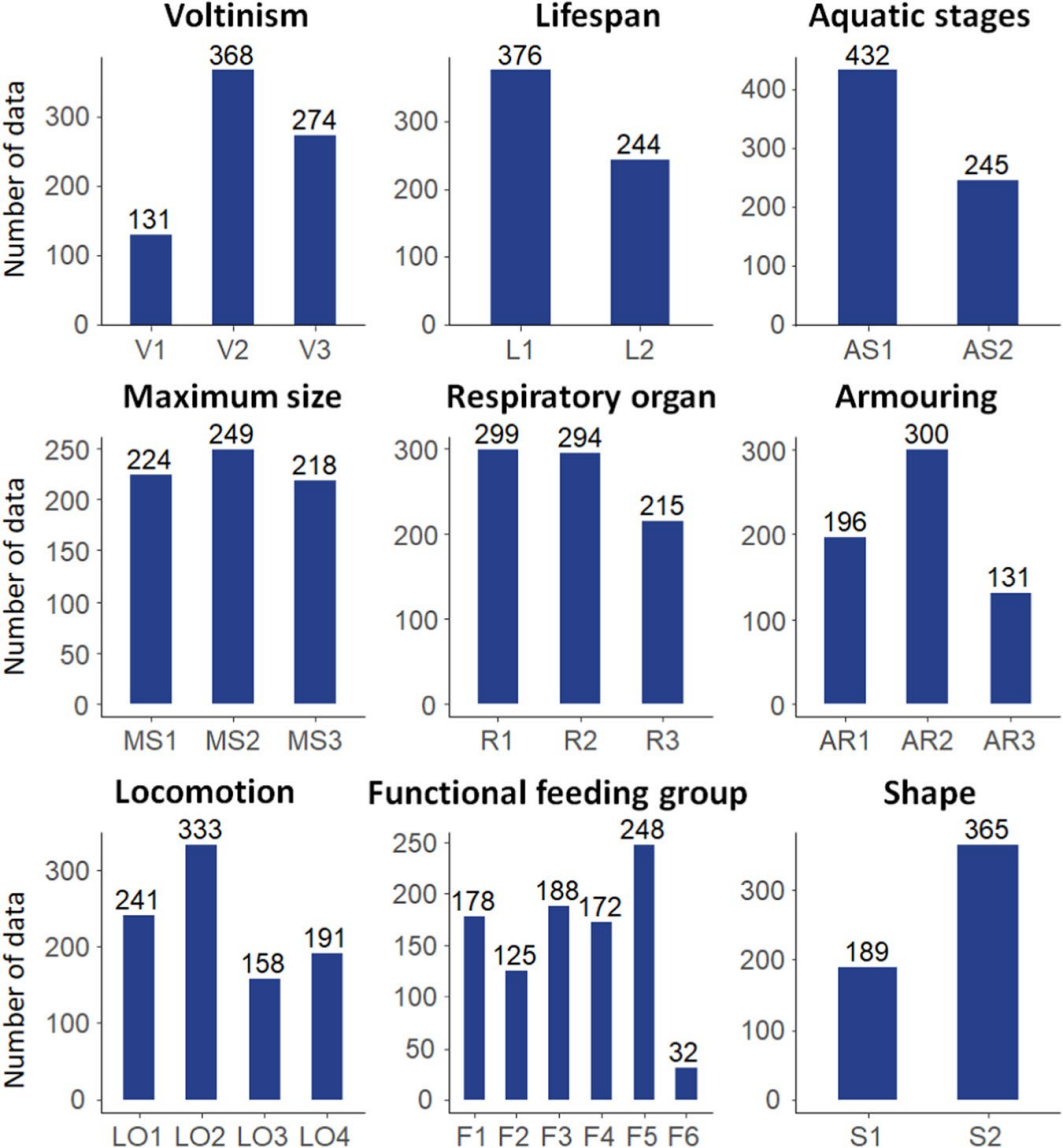
Summary of the traits in the dataset. The abbreviations are described in Table 1.

The dataset includes 447 taxa (393 at genus level, 53 at family level and one at class level) from five phyla. Arthropoda has the largest representation with 367 genera, followed by Mollusca (49 genera), Annelida (29 genera), Platyhelmenthes (3 genera), and Nematomorpha (2 genera). Of the 6,616 non-zero records, 24.14% are classified as having very low confidence (1), 49.18% have a moderate level of confidence (2), and 26.68% have a high level of confidence (3). See Fig. 2 for a summary of the different traits.

## Technical Validation

**Fig. 3:**
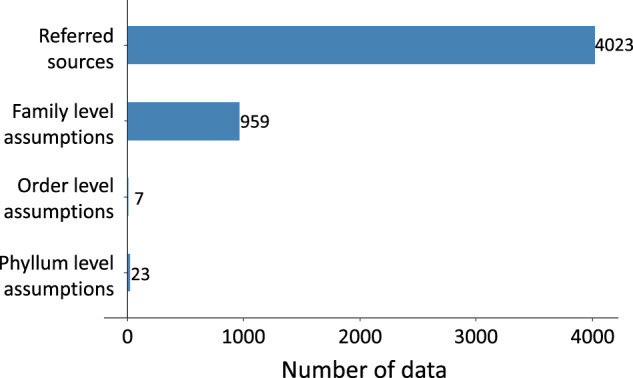
Sources of information in our dataset for different traits. The number of data indicates the number of trait modalities multiplied by the number of genera.

The biomonitoring data were collected through the NAEMP following the NIER guidelines^22^. Taxonomic experts identified all the specimens, and trait information was collected from a total of 154 sources, including journal articles, datasets, books, and web resources. To ensure accuracy, the resulting dataset underwent cross-checking for any mistakes. About 77% of the data in the dataset were sourced from the references, while the remaining 23% were inferred from higher taxonomic-level characteristics (Fig. 3). This indicates the dataset needs periodic updates to include trait data from more recent research.

## Usage Notes

The dataset we have compiled contains a wealth of information on new genera that have not yet been included in other existing trait datasets. As a result, it can help to fill some critical gaps towards developing an integrated global trait dataset. Our biomonitoring data consists of 51 endemic species belonging to 34 macroinvertebrate genera (*see* 'Korean endemic' sheet of the dataset^48^). While only one of these genera is endemic to Korea (*Koreanomelania*), the others share some species from other countries, particularly Japan and China. This broadens the applicability of the dataset and enhances its usefulness in different contexts.

This dataset provides a unique opportunity to better understand functional diversity, as well as the responses of different functional groups to environmental perturbations. It also enables researchers to compare similar functional groups at a global level, providing valuable insights into their effects on different stressors such as pollution and climate change.

The database uses fuzzy coding system to indicate probability of different traits. In this case, use of traits with higher confidence (2 & 3) are advised for application. The data is provided in an Excel workbook format (*.xlsx).

Lastly, this database is the pioneering effort to develop a functional trait dataset for streams & rivers of South Korea. It is still not comprehensive and many traits information are inferred from higher taxonomic levels due to lack of enough information. So, this dataset demands improvement via periodic updates to include more detailed information about the existing traits, to include additional traits, to increase the taxonomic resolution and to include the additional genera those are not yet included.

## Data Availability

The dataset is accessible from Figshare^48^.

## Code availability

No custom code has been used.
